# Oxidative physiology of two small and highly migratory Arctic seabirds: Arctic terns (*Sterna paradisaea*) and long-tailed jaegers (*Stercorarius longicaudus*)

**DOI:** 10.1093/conphys/coad060

**Published:** 2023-08-19

**Authors:** Melinda A Fowler, Joanna B Wong, Autumn-Lynn Harrison

**Affiliations:** Department of Biology/Chemistry. Springfield College, 263 Alden Street, Springfield, MA 01109 USA; Institute for the Oceans and Fisheries, University of British Columbia, Vancouver, BC, V6T 1Z4, Canada; Department of Bird Migration, Swiss Ornithological Institute, 6204 Sempach, Switzerland; Smithsonian‘s National Zoo and Conservation Biology Institute, Migratory Bird Center, 3001 Connecticut Avenue, NW, Washington, DC. 20008 USA

**Keywords:** migration, oxidative stress, seabirds

## Abstract

Arctic ecosystems are changing rapidly. The tundra supports nesting migratory seabirds that spend most of their year over the ocean. Migrations are demanding, but it is unclear how physiological capability may equip organisms to respond to their changing environments. For two migratory seabird species nesting in Alaska, USA, the Arctic tern (n = 10) and the long-tailed jaeger (n = 8), we compared oxidative physiology and aerobic capacity measured during incubation and we recorded individual movement paths using electronic tracking tags. Within species, we hypothesized that individuals with longer-distance migrations would show higher oxidative stress and display better aerobic capacity than shorter-distance migrants. We examined blood parameters relative to subsequent fall migration in jaegers and relative to previous spring migration in terns. We present the first measurements of oxidative stress in these species and the first migratory movements of long-tailed jaegers in the Pacific Ocean. Arctic terns displayed positive correlation of oxidative variables, or better integration than jaegers. Relative to physiological sampling, pre-breeding northward migration data were available for terns and post-breeding southward data were available for jaegers. Terns reached a farther maximum distance from the colony than jaegers (16 199 ± 275 km versus 10 947 ± 950 km) and rate of travel northward (447 ± 41.8 km/day) was positively correlated with hematocrit, but we found no other relationships. In jaegers, there were no relationships between individuals’ physiology and southward rate of travel (193 ± 52.3 km/day) or migratory distance. While it is not clear whether the much longer migrations of the terns is related to their better integration, or to another factor, our results spark hypotheses that could be evaluated through a controlled phylogenetic study. Species with better integration may be less susceptible to environmental factors that increase oxidative stress, including thermal challenges or changes in prey distribution as the Arctic climate changes rapidly.

## Teaser

We present the first oxidative physiology values of two species of conservation need, Arctic terns and long-tailed jaegers. Both migrate long distances after nesting on tundra, a habitat impacted by climate change. Terns exhibited better oxidative integration than jaegers, although physiology was not related to migration metrics within either species.

## Introduction

Climate change is altering the composition, structure and functioning of terrestrial and marine ecosystems in ways that may be abrupt and irreversible (IPCC, 2021). Arctic and alpine tundra are among the most rapidly changing terrestrial ecosystems (Post *et al.,* 2009; Anderegg and Diffenbaugh, 2015) and in the oceans, many species have already shifted their migration patterns and distributions (IPCC, 2021). Arctic seabirds link these two changing biomes through long-distance migrations from tundra nesting habitat to marine post-breeding habitats. Migration and nesting are physiologically demanding periods of avian annual cycles, but for many Arctic species, baseline information on organismal physiology remains unknown.

The physiological capability of an animal has the potential to dramatically affect the way it uses habitat, including foraging and reproductive decisions (Costa and Sinervo, 2004; Madliger *et al.,* 2018). Calls have been made for greater consideration of physiology in conservation, including the need to investigate how physiological metrics may assist in predicting organismal sensitivity to global change (Cooke *et al.,* 2021). As climate change continues to alter oceans and tundra habitats, such information is important for understanding how potential ecosystem changes may impact the health, foraging and reproductive success of animals like long-distance migratory seabirds that capitalize on both systems. Oxidative stress has been identified as a tool in the conservation physiology “toolbox” for predicting and monitoring responses to environmental change (Madliger *et al.,* 2018). It is understood that oxidative stress affects many aspects of an animal's health, but additional data are needed to predict how it may affect an animal's response to a changing environment (Beaulieu and Costantini, 2014; Cooper-Mullin and McWilliams, 2016; Skrip and McWilliams, 2016; Costantini *et al.,* 2019). Thus, quantifying oxidative status of organisms in relation to life stages is an important first step (Beaulieu and Costantini, 2014).

Oxidative stress is an imbalance between the damaging by-products of aerobic metabolism (reactive oxygen species, ROS) and the molecules that can protect cells from these damaging by-products (antioxidants) (Sies, 1991; Jones, 2006; Beaulieu and Costantini, 2014; Costantini, 2016) and is a consequence of requiring oxygen for aerobic metabolism. This requirement leads to the generation of oxidizing agents, collectively called reactive oxygen species (Finkel and Holbrook, 2000; Costantini *et al.,* 2010) that can damage cellular components including membrane lipids, proteins and nucleic acids (reviewed in (Costantini, 2016). However, antioxidant molecules can in turn, minimize or prevent oxidative damage (Costantini *et al.,* 2010; Cooper-Mullin and McWilliams, 2016). Antioxidants take both enzymatic and non-enzymatic forms and may include vitamins and uric acid, a byproduct of protein catabolism (Cohen and McGraw, 2009).

There are four contributors to oxidative stress: 1) the production of free radicals (unstable biochemical species with one or more unpaired electrons, 2) the damage they cause, including the production of subsequent molecules that further oxidative damage, 3) antioxidant conditions and 4) repair mechanisms (Costantini, 2008; Monaghan *et al.,* 2009). Measuring all four aspects is extremely difficult in free-ranging animals, and it is rare to find data on free radical production and repair mechanisms. ROS, in our study measured as organic hydroperoxides, are considered to be biomolecules that have been damaged by free radicals and reflect a measure of damage because they are the products of oxidative damage (Costantini, 2016). They may then also participate in an oxidative cascade by continuing to produce more free radicals, as well as damage other cellular components, such as lipids, proteins and nucleic acids (Costantini *et al.,* 2010; Skrip and McWilliams, 2016).

The components of oxidative stress are dynamic. Organisms experiencing fluctuations in the production of ROS may counteract with antioxidants, which can be obtained through diet, or produced endogenously (Cooper-Mullin and McWilliams, 2016). The dynamic nature of oxidative stress means that it may not be in balance at all times, and if ROS production is not matched by antioxidants, organisms may experience oxidative stress (Costantini and Verhulst, 2009; Monaghan *et al.,* 2009). The way in which these components interact can be evaluated through integration, which is the concept that the components of a biological system will demonstrate some inter-relationships related to functionality (Ravasz *et al.,* 2002; Cohen *et al.,* 2012). Integration is often viewed as a network and evaluated via correlation matrices of physiological variables (Mitteroecker and Bookstein, 2007; Cohen and McGraw, 2009). Comparing the inter-relationships between oxidative physiology components across species can help provide insight into the management of their oxidative physiology (Cohen *et al.,* 2012; Costantini *et al.,* 2013). Life history variables (reproduction, senescence, survival) can be affected if organisms are not adept at balancing resources (energetic or non-energetic) dedicated to addressing oxidative status (Alonso-Alvarez *et al.,* 2006, 2017; Monaghan *et al.,* 2009; Costantini *et al.,* 2016).

The exact time frame over which oxidative perturbations may be detected and linked to environmental or life history variables remains uncertain. Some studies have detected oxidative stress perturbations on the order of 18 to 24 hours (migrating bats (Costantini *et al.,* 2019)) to several weeks after thermal challenges (bats (Beaulieu *et al.,* 2020)) to years (lifespan in seabirds (Costantini and Dell’Omo, 2015)). Therefore, oxidative stress values may reflect acute challenges or be integrative of longer term challenges.

Multiple studies of breeding birds have demonstrated links between oxidative stress and current or future reproduction. Studies in songbirds have demonstrated disruptions in oxidative homeostasis relative to clutch size, lay date and provisioning effort (Guindre-Parker *et al.,* 2013; Costantini *et al.,* 2016; Fowler and Williams, 2017; Pap *et al.,* 2018). In seabirds, (Scopoli's shearwater and European shags) measurements of oxidative damage in the chick rearing period were linked to shorter lifespans and reduced survival (Costantini and Dell'Omo, 2015; Herborn *et al.,* 2016). In addition to the effect of reproduction, oxidative stress can also be affected by food availability (Crommenacker *et al.,* 2011), fasting (Schull *et al.,* 2016), aging, stress (Hau *et al.,* 2015), inflammation and exercise (Powers and Jackson, 2008; Nikolaidis *et al.,* 2012).

Aerobic capacity refers to potential for oxygen uptake (Bech and Klaassen, 1996). Oxygen transport involves many factors, including oxygen intake in the lungs, transport through the circulatory system and utilization by muscles (Wagner, 1996; Williams *et al.,* 2012). Red blood cells are critical, as they contain the pigment hemoglobin, which binds to oxygen to carry in through the vascular system to the tissues. Although not the only determinants of aerobic capacity, hematocrit or packed red blood cell volume (Yap *et al.,* 2018) and hemoglobin (Calbet *et al.,* 2006) are key determinants of oxygen transport and can affect the ability to sustain intense workloads (Williams, 2012), such as migration. These metrics can vary in birds, due to some combination of environmental effects and physiological variables, such as reproduction (Williams, 2012).

Organisms undergoing long distance migration may be at risk for increased oxidative stress (Cooper-Mullin and McWilliams, 2016). Fuel quality and antioxidant content before and during stopovers (periods of rest or refueling during migration) may affect oxidative stress levels (Pierce and McWilliams, 2014; Skrip and McWilliams, 2016). Migrating bats incur oxidative stress due to flight (Costantini *et al.,* 2019) and migrating birds, highly dependent on lipid to fuel their flight, may face an unavoidable cost of oxidative damage (Skrip *et al.,* 2015). The occurrence of oxidative damage and ability to access antioxidants could limit the flexibility of foraging/migration strategies (Jenni-Eiermann *et al.,* 2014). However, more data are needed to link oxidative stress to foraging or migration performance. In non-migratory species, flight performance has been linked to oxidative stress (Costantini, 2008; Larcombe *et al.,* 2008; Costantini *et al.,* 2013; DeMoranville *et al.,* 2022), but quantifying oxidative stress during migratory flight is challenging because animals are difficult to access mid-migration unless at a stopover site, especially for species that migrate over oceans. Studies in songbirds at their stopover sites showed increased antioxidants (blackbirds; (Eikenaar *et al.,* 2017)), increased oxidative damage (garden warblers; (Skrip *et al.,* 2015)) or both increased oxidative damage and antioxidants (European robins (Jenni-Eiermann *et al.,* 2014)). Conversely, Northern bald ibis showed no oxidative changes during flight (Bairlein *et al.,* 2015). Seabirds are underrepresented in field studies linking oxidative stress with movement metrics (foraging or migration), with only penguin oxidative stress linked to foraging (Beaulieu *et al.,* 2010; Colominas-Ciuró *et al.,* 2017, 2021).

In this study, we quantified oxidative stress and aerobic capacity of two species of highly migratory seabirds during the incubation period: long-tailed jaegers (*Stercorarius longicaudus,* ~ 350 g, also called long-tailed skuas) and Arctic terns (*Sterna paradisaea,* ~ 100 g*)*. We were also interested in relating individual migration metrics to their oxidative physiology and explored this by following the migrations and foraging movements of terns and jaegers over the Pacific and Southern Oceans using electronic tags. Both species are relatively small and highly acrobatic seabirds that make long-distance migrations to the Southern Hemisphere from their Arctic and sub-Arctic breeding habitats, have a circumpolar breeding distribution and in some places, breed sympatrically (Harrison *et al.,* 2021; Wong *et al.,* 2021). Both Arctic terns and long-tailed jaegers are listed as “species of greatest conservation need” within Alaska with Arctic terns being listed for every bioregion in the state (Alaska Dept of Fish and Game, 2015). Long-tailed jaegers are also considered by the State of Alaska to be both sentinel species (indicators of environmental change) and stewardship species (species with a high percentage of their North American or global populations in Alaska). We were interested in physiological measures of to inform multiple aspects of Arctic seabird conservation and management including 1) establishing first measures for the oxidative physiology of these two species to inform future biomarker monitoring in rapidly changing Arctic and ocean habitats; 2) collecting measures that could be incorporated into population health assessments in lieu of direct demographic information; 3) providing foundational analyses for generating broader hypotheses about the relationship between integration, migration capability and predicted responses to environmental challenges across seabird species.

Recent papers linking oxidative physiology to population health note that in situations where populations or ecosystems may be difficult to monitor, but sentinel individuals are relatively easy to catch, physiological measures could be bioindicators of population health if incorporated into conservation planning (Adams and Ham, 2011; Beaulieu *et al.,* 2013; Bergman *et al.,* 2019). No firm population estimates exist for jaegers, and for terns, population estimates for interior Alaska are especially deficient (U.S. Fish and Wildlife Service, 2009). Terns are known to redistribute colonies from year-to-year, or to skip breeding when conditions are poor (Egevang and Frederiksen, 2011; Mallory *et al.,* 2017; Scopel and Diamond, 2018). This diffuse nesting structure, and potential for redistribution, makes population monitoring difficult and expensive to obtain, even via a sampling approach, across the vast and remote Alaskan Arctic.

While most jaeger nesting habitat in Alaska is low-lying coastal tundra, there is an isolated inland breeding population of long-tailed jaegers in montane tundra in Denali National Park (where 6 of our 8 birds were sampled). Despite being located within a National Park, this population is difficult to survey due to the remote nature and large size of the Park, making the detection of declines or distribution shifts very difficult (McIntyre, 2007). Jaegers are important predators in the Arctic food web (Gilg *et al.,* 2003; Krebs *et al.,* 2003). They consume small mammals (voles and lemmings), shorebirds and songbirds. Structured interviews of those with 20 to 51 years of experience in the Park noted declines of long-tailed jaegers and American golden-plovers (a jaeger prey/host species) within the Park (Meeker *et al.,* 2022). Climate change effects, including elevation-dependent warming (Pepin *et al.,* 2015), include a pattern of woody plant encroachment on Arctic tundra in Alaska (Terskaia *et al.,* 2020; Raiho *et al.,* 2022), including moving higher in elevation to the montane tundra jaeger and shorebirds use as nesting habitat in the park. Thus, transition of tundra to shrub and woody vegetation, increased tourism, effects to prey species and general warming of the Arctic, all have the potential to disrupt both species’ nesting success and in turn, Arctic and sub-Arctic food webs. Seabirds in general are characterized by having low reproductive rates (Dobson and Jouventin, 2007) and are vulnerable to cumulative impacts that may reduce adult survival or otherwise decrease reproductive rates (Sydeman *et al.,* 2021). Given similarities in many aspects of the two species life histories, and shared threats to their nesting habitat, collecting baseline information of these species' physiological status during their nesting season is essential.

Away from nesting habitats, both species switch to an oceanic lifestyle. The migrations of Arctic terns include the longest recorded animal migration of 80 000 km round-trip (Egevang *et al.,* 2010) from the Arctic tundra to foraging grounds off Antarctica. Most populations in the Atlantic Ocean use the same migratory routes (Wong *et al.,* 2021) while in the Pacific Ocean, birds migrating from Alaskan breeding grounds largely stayed in the eastern Pacific Ocean during migration (McKnight *et al.,* 2013; Duffy *et al.,* 2014). No studies of the oxidative physiology of Arctic terns have been published, although Costantini (2008) speculated on the potential relationship between extreme migration, longevity and flight related oxidative stress in the species. The mitochondrial DNA of Arctic terns was recently sequenced (Skujina *et al.,* 2019) and may provide insight into metabolic adaptations, but no relationships have been elucidated as of yet.

Atlantic Ocean migration patterns of long-tailed jaeger populations are well studied. Birds from breeding grounds in the central Canadian Arctic (Harrison *et al.,* 2021), and Greenland and northern Scandinavia make annual migrations to west and southern Africa (Gilg *et al.,* 2013; van Bemmelen *et al.,* 2017) and can cover as much as 900 km/day (Sittler *et al.,* 2011). However, there are no migration studies of long-tailed jaegers that use the Pacific Ocean; distribution data show sightings as far south as New Zealand and Chile (Furness, 1987; Wiley and Lee, 2020). No information is currently available about long-tailed jaeger oxidative physiology.

Birds like Arctic terns and long-tailed jaegers that fly great distances may have a physiological oxidative cost, which may carry over into other life history phases; although the timing of these physiological signals’ persistence is uncertain. Our goals were to 1) establish baseline incubation oxidative stress and aerobic capacity of two seabird species that link the Arctic region to marine habitats through long migrations, but that we hypothesize differ in their migration distance, 2) evaluate the integration of each species’ physiological capacity and 3) test the hypothesis that within species, individual migration distance and rate of travel are positively correlated with their incubation oxidative stress.

## Methods

### Field methods

We captured adult Arctic terns and long-tailed jaegers in Alaska, USA during the late-incubation period of nesting (mid-June to early July 2018) with manually triggered bownet traps (Bub, 1991) set at nests. The eggs remained in the nest during processing and were not harmed. Typically, upon capture of one member of the nest, the mate would take its place incubating the eggs while we processed the captured individual. We recorded standard morphometrics (mass, flattened wing chord, diagonal tarsus, bill and total head plus bill), collected three breast feathers for DNA sexing (Animal Genetics, Tallahassee, FL, USA) and took blood samples (see below). All birds were fitted with a USGS Bird Banding Laboratory metal band.

Ten Arctic terns (four females, two males, four unknown) were captured in coastal tundra near Alpine, Alaska, USA. Full migration details are reported in Wong *et al.* (2021). Six individuals were previously captured in 2017 and tracked with 0.65 g archival light-level geolocators (Migrate Technology Ltd, Cambridge, UK, model W65). Tags were wound with self-amalgamating tape and attached to a Darvic PVC color-band on the leg with a miniature UV-resistant zip-tie. The final attachment weighed 1 g total mass (<2% of the body weight of the tern). Upon re-capture in 2018, we removed geolocators and color-bands, recorded morphometrics as described above and took feather and blood samples. We also newly captured and sampled an additional four individuals—these birds were banded with USGS metal bands upon capture but no previous tracking devices were carried by these birds, and no new devices were deployed. These birds were sampled to obtain additional baseline physiology measures.

Eight jaegers (four nesting pairs, four males, and four females) were captured, sampled and fitted with satellite tags. We captured six individuals in sub-Arctic alpine tundra habitat in Denali National Park and Preserve, Alaska, USA (63.1148° N, 151.1926° W) and two individuals in tundra habitat of the Arctic coastal plain near Alpine Alaska, USA (70.3281° N, 150.9775° W). The long-tailed jaeger nest from Alpine, Alaska had an unwitnessed depredation prior to capture; upon capture we first saw the nest contained only eggshell fragments. Both birds continued to exhibit incubation behavior by sitting on the nest as if eggs were still present. We attached 5-g Argos solar-powered satellite tags (Microwave Telemetry Inc. Columbia, MD USA; actual tag mass, 4.48–4.57 g; mean = 4.5 g) to the birds using a leg-loop harness (Mallory and Gilbert, 2008) made of either 4.7625 mm tubular Teflon (n = 5) Ribbon or 2.54 mm Spectra (n = 3)), both from Bally Ribbon Mills, and secured with aluminum crimps. Tags were duty cycled to transmit for 10 hours, and to charge for 48 hours, but were also programmed to remain transmitting when fully charged and thus often transmitted daily. The final attachment weighed a mean of 4.8 to 6.0 g (1.7–2.1% of the body weight of the birds). We assessed wing and leg mobility prior to release. These tags are not recovered and remain on the bird until the harness material degrades, the tag is shed due to weight changes, the tag stops functioning or charging, or the bird dies. Tags often cease transmission over ocean and fate is typically unknown.

We sampled blood (~ 0.5 mL) from the brachial vein and collected into a heparinized microcentrifuge tube after birds were fitted with bands and tags and prior to release. Samples were stored on ice until return to the field station. Upon return to the field station, we measured hematocrit, aliquoted whole blood to hemoglobin reagents (see below) and separated plasma by centrifuge (LW Scientific ZipCombo) at 12 000 rpm for 5 minutes. Plasma samples were frozen at −20°C until shipped on dry ice, where they were stored at −80°C until laboratory analysis. Hemoglobin samples were protected from light and refrigerated until analysis. Processing time from capture to blood sampling prior to release ranged from 32 to 60 minutes and we assessed the effect of holding time on oxidative variables by looking for correlations between oxidative physiology variables and time to sampling. There is variability in previous studies regarding the effect of restraint on oxidative variables. In domestic chickens, antioxidants in some birds increased dramatically after 1 hour of restraint, but in other birds decreased dramatically (Cohen *et al.,* 2007). Oxidative stress appears to be stable (no variation) for over 30 minutes of restraint based on studies of barn swallows, garden warblers, snow buntings and house sparrows (Costantini *et al.,* 2007; Guindre-Parker *et al.,* 2013; Huber *et al.,* 2017). Restrained bats showed no change over 77 minutes of restraint (Beaulieu *et al.,* 2020). King penguins showed variation in oxidative stress after 30 minutes of capture, but overall did not support the idea that acute stress has strong effects on oxidative damage (Stier *et al.,* 2019).

After release, we monitored birds visually to assess flight and monitored the return to the nest. All nests continued to be incubated after capture with the exception of the jaeger nest that had been depredated prior to capture. We do not have nest success (hatch and fledging) for most nests because we did not want to disturb the nest site further and in some cases we departed the field site prior to hatch.

Trapping, handling, marking and processing birds was done under permits from the Alaska Department of Fish and Game (Alaska Scientific permit 17-162 and 18-156 to A.-L. Harrison), the U.S. Geological Survey Bird Banding Laboratory (permit 09700 to P.P. Marra). Animal care protocols were approved by the Institutional Animal Care and Use Committees of the National Zoological Park (protocol 15-33 and 18-06, P.I. A.-L. Harrison) and the National Park Service (AKR_DENA_Harrison_Jaegers_2018.A2, P.I. A.-L. Harrison).

### Laboratory analyses

We analysed a set of variables indicative of aerobic capacity (hematocrit and hemoglobin), oxygen metabolites (plasma reactive oxygen metabolites) and antioxidants (plasma non-enzymatic antioxidant levels and plasma uric acid). We measured hematocrit (% packed cell volume), hemoglobin (g·dl^−1^), plasma non-enzymatic antioxidant levels (μmol HClO mL^−1^; OXY), plasma reactive oxygen metabolites (mg H_2_O_2_ dL^−1^; ROM) and plasma uric acid (mg∙dL^−1^) as described in (Fowler *et al.,* 2018). Briefly, hematocrit was measured following centrifugation for 3 minutes at 7500 *g*, using digital calipers (± 0.01 mm). We then determined the percentage of packed red cell volume to total column height (plasma plus packed red cell volume). Sample size for hematocrit: terns n = 10, jaegers n = 8. We measured hemoglobin (g∙dL^−1^ whole blood) using the cyanomethemoglobin method (Drabkin and Austin, 1932) modified for use with a microplate spectrophotometer, using 5 μL whole blood diluted in 1.25-mL Drabkin’s reagent (D5941 Sigma-Aldrich) with absorbance measured at 540 nm (following (Wagner *et al.,* 2008b)). Pointe Scientific standards were used (H7506-STD). Mean intra-assay coefficient of variation was 1.8% and mean inter-assay coefficient of variation was 1.3%. Sample size for hemoglobin: terns n = 10, jaegers n = 7. Uric acid (mg∙dL^−1^) was measured with the Bioassay Quantichrom assay (DIUA-250) following the manufacturer’s guidelines. Samples were assayed in duplicate using 5-μL sample volume following 1:3 dilution with dd H_2_O. All samples were measured in one plate and mean intra-assay coefficient of variation was 4.9%. Sample size for uric acid: terns n = 8, jaegers n = 7.

For plasma reactive oxygen metabolites and plasma non-enzymatic antioxidants, we used oxidative stress assays that have been previously validated for avian sampling (Costantini, 2016; Skrip and McWilliams, 2016). Reactive oxygen metabolites (mg H_2_O_2_ dL^−1^; ROM) were measured using the dROM kit from Diacron International, Italy (catalog number MC001), modified after (Guindre-Parker *et al.,* 2013). All samples were measured in one plate and mean intra-assay coefficient of variation was 4.4%. Sample size for ROM: terns n = 8, jaegers n = 6. Total non-enzymatic antioxidant titers (μmol HClO mL^−1^; OXY) were determined using the OXY kit from Diacron International, Italy (catalog number MC434), modified after (Guindre-Parker *et al.,* 2013). All samples were measured in one plate and mean intra-assay coefficient of variation was 7.8%. Sample size for OXY: terns n = 9, jaegers n = 7. Uric acid’s antioxidant capacity is not captured by the OXY assay and thus it is not “double” counted. The OXY assay does not need to be corrected for uric acid levels, it is distinct from the antioxidant capacity of uric acid (Costantini, 2011).

### Movement path estimation

We used movement models that account for the error associated with each tag type to convert raw data collected by electronic tags into location estimates. Differences in tag types necessitated different methods for processing their movement data. Light-level and sea-surface temperature data recorded by geolocator tags carried by Arctic terns were processed using the R statistical computing program (R Core Team, 2018) packages *TwGeos* (Lisovski, 2016) and *SGAT* (Wotherspoon *et al.,* 2013); full details in (Wong *et al.,* 2021). When light-level is the same across the globe (for example, during periods of the equinox), or does not vary during a day (for example, during periods of 24-hour daylight at the poles), locations cannot be resolved. Therefore, Arctic tern migration paths are truncated; movements in the Arctic Circle and a period of the Southern Ocean residency for some individuals were unavailable.

Argos satellite data for long-tailed jaegers were processed using a continuous-time movement model (Jonsen *et al.,* 2020) following details in (Harrison *et al.,* 2021) and implemented in package *foieGras* for R.

Differences in sampling frequency of electronic tags can greatly affect distance estimates of a movement path (Noonan *et al.,* 2019). Because we were interested in comparing migration distances, we standardize model predictions to one position per day across tag types (the finest time frame available for geolocator devices).

### Timing of physiological sampling relative to timing of migration data

This study was part of a larger study quantifying migration patterns in tundra breeding birds. Due to field logistics for the larger study goals, we had limited control over the sampling opportunities relative to tag deployment or recovery. Although both species were sampled for physiological variables during the same year and breeding stage (2018, incubation), the timing of physiological sampling relative to the timing of tag attachment differed between species. For Arctic terns, physiological sampling occurred during a recapture-event when archival tags were removed from the bird and the data were downloaded from the tag. Thus, the movement record is from the year prior to physiological sampling. For long-tailed jaegers, samples were taken at time of satellite tag attachment (which then transmitted remotely indefinitely and was not removed in the future) and we recorded movement data during the year after physiological sampling. For each species, we chose the migration period closest in time to physiological sampling to relate individual physiology with individual migration metrics. For terns we analysed the northward (spring) portion of the migration track, just prior to its arrival at the breeding colony. Thus, relationships between migration metrics and physiology may reflect the effect of pre-breeding migration on breeding physiology this species. For jaegers we analysed the southward migration, after the bird left the breeding colony in the fall, allowing us to evaluate the effect of breeding physiology on post-breeding migration. Many bird species differ in their rates of travel and migration distances between the northbound and southbound migrations due to seasonal variation in the environment (for example, prevailing winds or environmental perturbations such as El Niño Southern Oscillation) and timing of key life history events relative to the migration (for example, migrating to select a nest site and find a mate vs. migrating after breeding). In Arctic terns, for example, the pre-breeding migration was faster and more direct than the post-breeding migration in the Pacific Ocean (Wong *et al.,* 2021) which could have different effects on tern physiology. Given that we analysed different migratory periods for the two species, we could not make comparisons between the species regarding the relationship between individual physiology and their migration metrics.

### Migration metrics

**Figure 1:**
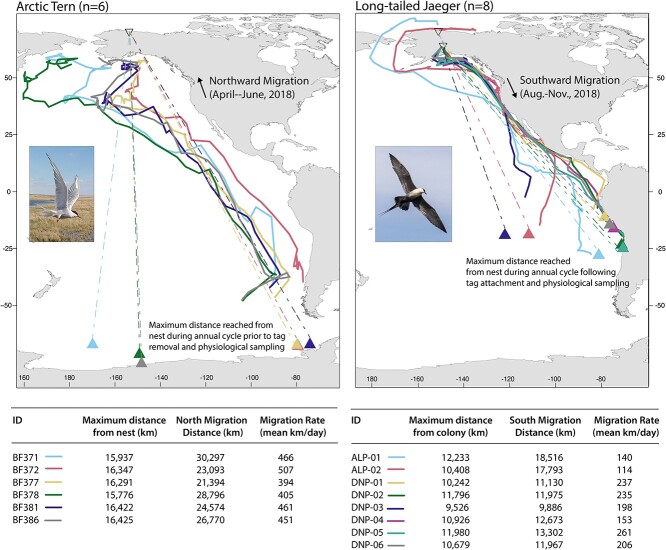
Migratory metrics used to relate to physiological variables measured during incubation for two seabird species tracked with electronic tags from nesting grounds in Alaska, USA. Lines indicate periods of migration (fast, directed movement omitting breeding and over-wintering periods, but including migratory stopovers). The northward, pre-breeding migration of Arctic tern and southward, post-breeding migration of long-tailed jaeger are shown. Triangles indicate the location at which the bird reached a straight-line maximum distance from their nest site in the tracked year. See Supplementary Material, Fig. S1 for full-annual cycle movement paths.

Complete, full-annual-cycle movement paths were available for jaegers (satellite-tracked), but not for terns (Figure 1, note dotted line portions of tern movement paths) due to limitations of light-level geolocators discussed above. We selected movement metrics that could be estimated from both technologies: 1) maximum distance reached from colony, 2) the distance of the migratory portion of the movement path, 3) rate of travel of the migratory portion of the movement path (migration distance divided by the time to cover the distance).

Maximum distance from colony was calculated as the farthest distance recorded by each bird from the known nest site. For Arctic terns, although a portion of their non-breeding period was unavailable due to 24-hour polar sun, positions in the Southern Ocean were documented for all individuals and relative differences between terns and jaegers were apparent (see Results).

To estimate the distance and rate of travel of the migratory portion of the movement paths, we first needed to delineate that portion. For terns, the migratory portion of the pathway was previously calculated in (Wong *et al.,* 2022). For long-tailed jaegers, we applied a change point analysis to a time series of net squared displacement to segment the track into breeding, migratory and non-breeding periods of the annual cycle. Net squared displacement (NSD) is the distance from colony of each location, squared and has been shown to increase rapidly during periods of fast, directed travel like migration behavior (Bunnefeld *et al.,* 2011). When the bird is resident to a place (in a home range or stationary period, as in a breeding location), NSD plateaus. We used the *geodist* package in R to calculate NSD using the square of vincenty distances from colony, estimated for an ellipsoid Earth. A change point analysis, implemented with the *changepoint* package in R, identifies changes in the rate of change of NSD and suggests segment breaks, which we then manually reviewed and assigned to a period of the annual cycle based on date. A worked example is shown in Supplementary Material, Fig. S1. Brief stopovers during migration were included in the migratory segment. For each migration segment, we calculated the migration distance as the sum of vincenty distances between each daily location. We estimated the daily rate of migration as the migration distance divided by its duration.

### Statistical treatments

Normality and equality of variance between species were assessed for all variables (see Table 1 for list), and the R statistical computing program (2018) used for statistical analyses.

**Table 1:** Mean (±standard deviation) values of physiological variables and migration metrics measured in incubating Arctic terns (n = 10) and long-tailed jaegers (n = 8) captured in Alaska, USA

	**Arctic tern**	**Long-tailed jaeger**
**Mass** (g)^*^	98.9 ± 8.9	303.9 ± 17.3
**Hematocrit** (%)	45.7 ± 5.2	46.6 ± 4.7
**Hemoglobin** (g.dl^−1^)	14.1 ± 2.7	14.8 ± 3.2
**Uric acid** (mg.dL^−1^)	36.5 ± 17.0	27.8 ± 14.9
**Antioxidants** (OXY, μmol HClO ml^−1^)	291.6 ± 35.4	262.2 ± 55.8
**Reactive Oxygen Metabolites** (ROM, mg H_2_O_2_ dl^−1^)	2.7 ± 1.0	1.0 ± 0.4
**Wing chord** (mm)^*^	273.7 ± 6.2	305.1 ± 9.6
		
**Max distance from colony** (km)^*^	16 199 ± 275	10 947 ± 950
**Migration only distance** (km)	25 821 ± 3417^	13 405 ± 3107^#^
**Rate of migration travel** (km/day)	447 ± 41.8^	193 ± 52.3^#^

^*^indicates statistically significant difference between species (p < 0.001). Physiology samples: Arctic terns (n = 10), long-tailed jaegers (n = 8). Migration metrics: Arctic terns (n = 6), long-tailed jaegers (n = 8). ^: spring migration #: fall migration

To test for differences in baseline physiological values between species, we adjusted for mass by including it as a covariate in ANCOVA, after (Packard and Boardman, 1999) and (Skrip *et al.,* 2015).

To explore relationships among physiological variables within species as a measure of integration, we used Pearson’s correlations to create correlation matrices. After identifying significant correlations of physiological variables (p < 0.05) within species, we further explored using linear regression to extract slopes and include mass as a covariate. We also used principal components analysis (prcomp in R) to combine physiological variables into a composite for exploring relationships to migration metrics (after (Hõrak and Cohen, 2010)). For some birds (terns, n = 3; jaegers, n = 2), low blood volume prevented us from performing all assays on all individuals. Because PCA requires all individuals to have values for all variables, these individuals were omitted from analysis resulting in a final sample size for PCA of terns n = 7 and jaegers n = 6.

To compare maximum distances between species we used a Welch's ranked t-test (maximum distances were not normally distributed, nor were variances equal between the groups). Within species, to test the hypothesis whether migration distance or rate was related to oxidative stress we used linear regression with mass as a covariate. Additionally, we explored whether the extracted principal components were related to migration variables. For Arctic terns, there was only 1 nest from which both birds were sampled for physiology values. For all other nests, the individual is the only representative in our dataset for the nest, thus nest ID is not included as a covariate. For jaegers, we sampled physiology variables from all individuals from all 4 nests, however we do not have a sample size that will permit the addition of nest ID as a covariate.

## Results

**Figure 2:**
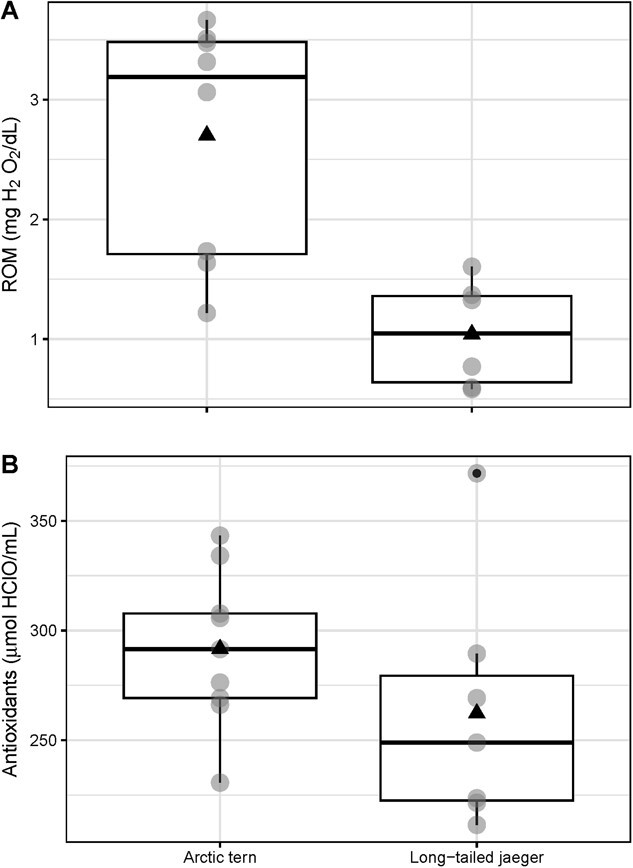
Variability in oxidative physiology between species. A: Reactive Oxygen Metabolites (ROM), B: Antioxidants (OXY) and C: Arctic terns and long-tailed jaegers captured in Alaska, USA. Horizontal lines indicate the median. Triangle (▲) indicates the mean. Apparent differences between distribution of raw values are not significant when mass is included as a covariate (see Table 1).

We report the first values of incubation oxidative stress and aerobic capacity variables in Arctic terns and long-tailed jaegers. When accounting for differences in mass between terns and jaegers, there were no statistically significant differences in any physiological variables between species (Table 1, Figure 2). The maximum distance recorded from breeding colony (year before sampling for terns, year after sampling for jaegers) was significantly farther for Arctic terns (16 199 ± 275 km) than for long-tailed jaegers (10 947 ± 950 km) (Table 1; Figure 1, Welch's ranked *t*-test t = 6.97, df = 16, p < 0.001). Additionally, terns were significantly lighter in mass (t = −30.5, df = 9.9, p < 0.001, 98.9 ± 8.9 g vs 303.9 ± 17.3 g) and had significantly smaller flattened wing chords (t = −8.0, df = 11, *p* < 0.00, 273.7 ± 6.2 mm vs 305.1 ± 9.6 mm) and mass and wing chord were highly correlated (*R* = 0.91, *p* < 0.001). Although the migration tracks were not compared statistically between species due to the different timing (northward vs southward), values for migration-only distance and rate of travel duration migration are reported in Table 1.

### Integration and relationships among physiological variables:

There was no significant relationships between physiological measures and our range of handling times (32–60 minutes) prior to sampling, or between sexes or nest ID. We therefore did not include sex or nest parameters in our models. There were notable differences across species in correlation patterns. Within jaegers, there were no significant correlations between physiological variables (Table 2). In contrast, within terns, antioxidants (OXY) were significantly and positively correlated with ROM and uric acid (Table 2). Because the correlation matrix (Table 2) indicated that different species had different inter-relationships of some variables, we used linear regression to look closer at how the variable relationships differed in the species.

**Table 2:** Correlation coefficients of physiological variables measured for incubating Arctic terns and long-tailed jaegers captured in Alaska, USA. (italics/bold = p < 0.05)

	**Arctic tern**				**Long-tailed jaeger**			
	Hb	OXY	ROM	Uric Acid	Hb	OXY	ROM	Uric Acid
Hematocrit	0.57	−0.14	−0.23	−0.50	−0.29	−0.54	−0.13	−0.62
Hemoglobin (Hb)		0.18	−0.11	−0.19		0.09	0.34	−0.08
Antioxidants (OXY)			***0.86***	***0.90***			0.42	0.69
Reactive oxygen metabolites (ROM)				***0.95***				0.78

**Figure 3:**
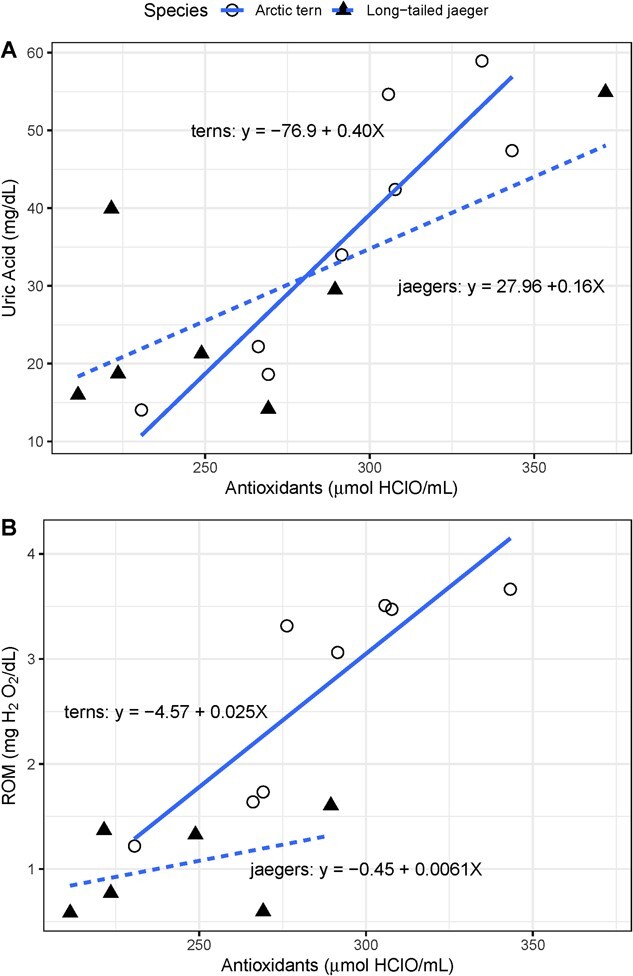
A) Relationship between physiological variables measured in incubating Arctic tern and long-tailed jaegers captured in Alaska, USA. Within terns, uric acid and antioxidants were significantly and positively related (adjusted R^2^ = 0.74, F_2,5_ = 10.76, p = 0.02), while in jaegers, there was no significant relationship. B) In terns, antioxidants were significantly related to ROM (reactive oxygen metabolites) (adjusted R^2^ = 0.64, F_2,5_ = 7.2, p = 0.03), but not in jaegers.

We found different relationships between uric acid and OXY in the different species, however the correlative approach does not account for animal mass. We thus further evaluated this relationship using linear regression with mass as a covariate. We found antioxidants were significantly related to uric acid in Arctic terns (*F*_(2,5)_ = 10.76, *p* = 0.02, adjusted *R*^2^ = 0.74, Figure 3A). In contrast, long-tailed jaegers did not exhibit a significant relationship between uric acid and OXY when mass was included as a covariate. We explored removing a potential outlier, the high jaeger OXY value (371 μmol HClO∙mL^−1^), but strength of the relationship decreased even further, confirming the lack of relationship between jaeger OXY and uric acid.

Although mean OXY and ROM values were statistically equivalent between species, within species they were significantly correlated in terns only (Table 2). Within each species, with mass included as a covariate, OXY significantly predicted ROM for terns (*F*_2,5_ = 7.2, *p* = 0.03, adjusted *R*^2^ = 0.64, Figure 2B), but not jaegers.

To further explore the differences in integration indicated by the previous analyses, we performed principal components analysis within each species. In terns, the first component accounted for 59.1% of the variation, and the second component accounted for 34.1%. Uric acid (−0.58), ROM (−0.56) and OXY (−0.52) loaded negatively, while Hb and Hct loaded positively. For jaegers, the first component carried 40% of the variation and the second 26.6%. The relationships among oxidative variables were not as clear or as strong in jaegers compared to terns. ROM loaded strongly (−0.68), as did uric acid (−0.53), but OXY did not and Hb was the third highest loading (−0.31), in the opposite direction of the terns.

### Relationship between migration distance and physiology

**Figure 4:**
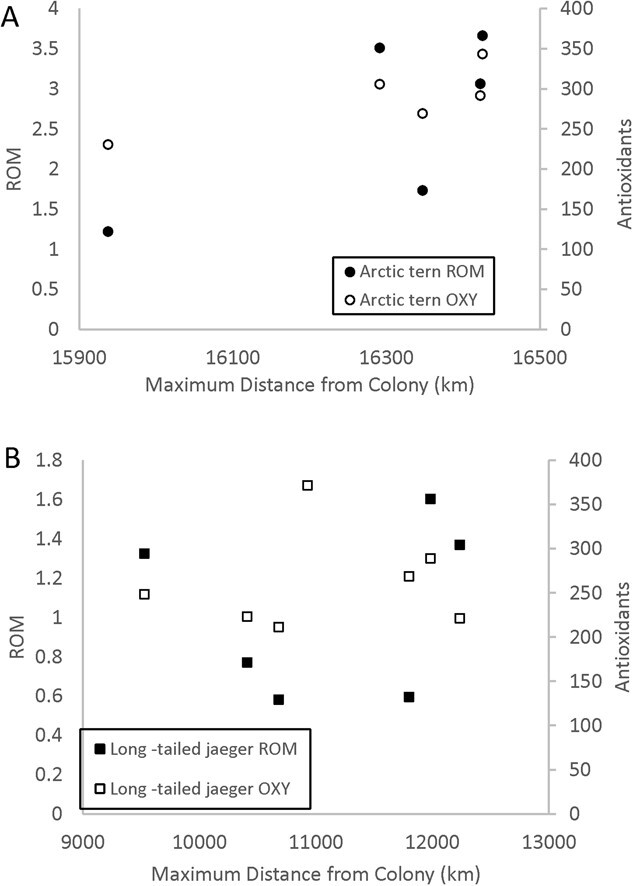
Maximum distance from colony is not related to either OXY or ROM. Individual OXY and ROM matched samples at the individual maximum distance from the colony.

For Arctic terns, there were no significant relationships between maximum distance individuals reached from colony and oxidative physiology (*p* > 0.05, Figure 4A). During their northbound spring migration prior to physiological sampling, the birds covered 25 821 ± 3417 km over a duration of 58.1 ± 9.1 days. The rate of travel was 447 ± 41.8 km/day (Table 1). Spring migration distance was also not related to tern oxidative physiology. However, we found a significant positive relationship between spring rate of migration (distance traveled/day) and hematocrit when controlling for mass (whole model: *F*_(2,3)_ = 10.7, *p* = 0.04. For hematocrit alone: *F*_(1,3)_ = 20.6, *p* = 0.02, *y* = 105.3 + 5.9x. Mass was not a significant predictor). When the first principal component of all the tern physiological variables was extracted and used as a variable for comparison to migration metrics, there was no relationship to distance or rate of travel (*p* < 0.05). Similarly, the extracted first principal component for Arctic tern ‘oxidative variables only’ was not related to migration distance or rate of travel (*p* < 0.05).

For long-tailed jaegers there were no significant relationship between maximum distance reached from colony and oxidative physiology (*p* > 0.05, Figure 4B). There was a significant negative relationship between jaeger hemoglobin and maximum distance, but one high hemoglobin value (21.7 g∙dL^−1^) drove the relationship, which disappeared when the outlier was excluded. Extracted principal components from all jaeger physiology variables showed no relationship to maximum distance reached from colony (*p* > 0.05). Additionally, we explored collapsing only the oxidative stress metrics (OXY, ROM and uric acid) for long-tailed jaegers. When ‘oxidative status variables only’ were used, the first component explained 63.1% of the variation and the second 33.3%. However, when we assessed the extracted components for relationships to maximum distance, none were found (*p* > 0.05). During their southbound fall migration following physiological sampling, jaegers flew 13 405 ± 3107 km over 78.5 ± 42.5 days, resulting in a travel rate of 193 ± 52.3 km/day (Table 1). Neither the individual variables, nor the extractions from the PCA showed a relationship to any physiology metrics. Similarly, the fall (southbound) rate of migration (distance traveled/day) was unrelated to the physiological variables we sampled.

## Discussion

Arctic Terns and long-tailed jaegers undertake physiologically demanding migrations of tens of thousands of kilometers each year (Egevang *et al.,* 2010; Harrison *et al.,* 2021; Wong *et al.,* 2021). In this study, we established first measures for the oxidative physiology of these two species to inform future biomarker monitoring of population health in the context of rapidly changing Arctic and ocean habitats. We provided foundational analyses for generating broader hypotheses about the relationship between integration, migration capability and potential responses to environmental challenges across seabird species. Contrary to our predictions, individuals that traveled farthest from their breeding site did not have higher individual oxidative stress during incubation for either species. Although our relatively small sample size does not permit generalizable inferences, our results suggest that Arctic terns may have better integration than long-tailed jaegers. Oxidative stress occurs when ROM increase, but antioxidants do not increase concomitantly (Costantini and Verhulst, 2009; Monaghan *et al.,* 2009). In captive zebra finches undergoing high intensity exercise, a reduction in biochemical integration is likely linked to a reduced resistance to oxidative stress (Costantini *et al.,* 2013). Although only an experimental manipulation would fully indicate responsiveness, we interpret the current correlation of ROM and antioxidants in terns to indicate an ability to avoid oxidative stress. Species with better integration capabilities may be better prepared to manage their response to oxidative challenges, while species with less adept integration, such as long-tailed jaegers, have the potential to be more severely impacted by scenarios that could trigger more oxidative stress, such as increased temperatures and habitat and prey availability fluctuations due to climate change.

For our sample of individuals from two breeding species, mass-corrected physiological values were statistically similar across species. Within each species, we found more correlations among physiological variables, indicating more integration, in terns compared to jaegers. Arctic terns showed a stronger positive relationship between their antioxidants and ROM, than did long-tailed jaegers. Terns also demonstrated a significant positive relationship between antioxidants and ROM, whereas the positive pattern we found in jaegers was not significant. Additionally, there was a significant positive relationship between tern uric acid and other antioxidants (OXY). These relationships were not present in jaegers. Uric acid is a by-product of protein metabolism, but also serves an additional antioxidant function (Tsahar *et al.,* 2006; Cohen and McGraw, 2009).

Dietary protein intake can affect uric acid values (Alan and McWilliams, 2013), but we do not feel that these integration pattern differences are due only to dietary differences. While we do not have specific diet for these individuals, generally, long-tailed jaeger breeding success is linked to their small rodent prey, including several species of voles and lemmings, their primary prey during the nesting season (Gilg *et al.,* 2003; Barraquand *et al.,* 2014). Conversely, Arctic terns eat primarily fish (Pratte *et al.,* 2017). Collared lemming protein content (dry matter) is ~ 57% (Batzli and Esseks, 1992) and sand lance, a possible prey item for terns (Robertson *et al.,* 2014) are 61–62% protein (Robards *et al.,* 2000). Thus, although Arctic terns and long-tailed jaegers have different prey types, the general protein content is likely to be similar. This is reflected in the statistically similar baseline uric acid values between the species. We interpret the additional positive correlations between uric acid, OXY, and ROM in Arctic terns to indicate that they may be responding more effectively to the generation of ROM.

In addition to establishing baseline incubation physiological values, a goal of our study was to investigate whether differences in migration metrics were related to oxidative stress patterns. Neither species demonstrated a relationship between individual oxidative physiology and the migration metrics we evaluated. We note that within species, differences in migration distances across individuals are small compared to the absolute differences in migration distances across species (Table 1) and that the longer distance migrant, Arctic terns, had higher integration among oxidative physiology variables.

There are, however, multiple caveats in interpreting the differences we observed across species. While our small sample size limits generalizability of our conclusions, these first values suggest interesting hypotheses to explore in future studies with larger samples. The two species we studied are within the same order (Charadriiformes, (Chesser *et al.,* 2021)), but are not within the same family. Garland and Adolph (1994) and Garland *et al.* (2005) caution against inferring evolutionary adaptations in studies comparing only two species and we cannot suggest that the difference in oxidative physiology we observed is an evolutionary adaptation to the much longer absolute migration distances of terns. There are not enough data on oxidative stress in different families of seabirds to rule out a phylogenetic influence on the patterns we describe. Sampling of additional families in this order across a range of migratory strategies and analysed with a phylogenetically independent contrast approach would aid in interpreting whether phylogeny and/or migratory strategy is a factor in explaining the oxidative physiology patterns we observe. The difference we found between species in integration is interesting and useful for helping us identify species more vulnerable to oxidative challenges, even if we do not yet know why the difference exists.

In migrating songbirds, fat mass has a positive relationship to ROM, and we encourage future studies to account for mass in oxidative studies, after (Skrip *et al.,* 2015). The two species in this study differed significantly in mass (Table 1). We accounted for mass by including it as a covariate in our linear analyses (Packard and Boardman, 1999). After this treatment, there was no statistical significance of oxidative physiology variables differing between groups. Whether mass differences between the species is a major factor beyond our statistical controls is unknown. We do not have specific lipid mass, but within each group, mass and ROM were not correlated. Additionally, the birds in this study displayed the opposite trend as (Skrip *et al.,* 2015)—smaller birds, terns, had higher ROM (Table 1). It seems unlikely that the higher mass of jaegers would be obscuring patterns—the expectation would be that the larger-bodied bird would have higher ROM simply from having more lipid to be oxidatively damaged. Instead, we found that smaller birds (terns) generated more ROM, and the correlative patterns of OXY and uric acid as antioxidants may be a response to that.

Another challenge to address is the temporal resolution regarding the time frame between sampling in incubation and migratory travel. How long would a physiological signal persist that would be relevant to indicate a relationship between a migratory trip they completed prior to breeding? Or to affect their post-breeding migration? Point samples of oxidative stress during the breeding season has been linked to future fecundity and survival in several avian species (Costantini and Dell’Omo, 2015; Herborn *et al.,* 2016; Fowler and Williams, 2017) and in bats, oxidative stress signatures are detectable two weeks after a thermal challenge (Beaulieu *et al.,* 2020), indicating that it is possible for signals to persist several weeks or months removed from the sampling time. The limits of temporal resolution for linking oxidative stress to other variables is an area physiological ecologists continue to explore. The better integration in Arctic terns, as indicated by correlations among ROM, antioxidants and uric acid (Costantini *et al.,* 2011, 2013), may indicate the capacity for greater responsiveness to oxidative challenges encountered in migration, although we cannot control for all variables. Increased oxidative stress may be an inevitable consequence of migration (Cooper-Mullin and McWilliams, 2016) and the tighter relationship between non-enzymatic antioxidants and uric acid may indicate the potential to counteract increased ROM as a consequence of flight. Reproduction may also impact oxidative stress (Alonso-Alvarez *et al.,* 2017), thus we cannot rule out that the better integration may also reflect the species’ differing responses to reproductive effort. While our samples are from incubation, we cannot be certain how well they reflect the level of oxidative stress seabirds experience mid-migratory flight. We did not find that the prior migratory effort, as we quantified it, in individual birds carried over to the oxidative stress in their breeding season (represented by the Arctic tern northward spring migration relative to their incubation physiology). We also did not find that individual oxidative stress measured in the incubation phase seemed to affect the individual migratory effort expended post-breeding, as reflected in long-tailed jaegers. In other species, mechanisms regulating integration capacity may persist across different life history stages and that integration may differ among species and be responsive to selective pressures (Costantini *et al.,* 2013). It is unknown whether the correlation between ROM and antioxidants displayed in incubation in Arctic terns would persist to other situations. Only experimental trials manipulating the exercise intensity would be able to clarify whether the integration in terns persists. However, the positive relationship between ROM and antioxidants in Arctic terns (indicative of a managed response to oxidative stress) (Costantini and Verhulst, 2009), may give some indication that they have the capacity to respond to future situations (such as migration or thermal stress) that may increase ROM levels with matching increases in antioxidants. Although without an experimental manipulation of ROM levels and measurement of the concomitant antioxidant response, we cannot definitively predict how jaegers might respond to a challenge increasing ROM, the lack of a correlation between ROM and antioxidants in the current study does not provide strong evidence that if ROM were to increase, that antioxidants would increase as well.

Our work informs the discussion of the temporal resolution for detecting individual oxidative stress signatures and their relationship to individual migratory performance metrics. As the field of wildlife oxidative physiology develops, timing of sampling is an area that is crucial to refine (Costantini, 2019). Individual relationships to migratory metrics (maximum distance, migration distance or migration rate) were not detected in our sample and across the range of migration distances exhibited within species. The individual variability of migratory metrics, relative to the overall distance traveled was not high, which may be an additional factor in an ability to detect relationships at the scale of inference. The temporal resolution of sampling is a variable that must be considered as physiological ecologists continue to refine timing of sampling necessary for detection of patterns relative to behavioral variables.

Plasma is a commonly used matrix for field studies of avian oxidative stress ((Costantini, 2016) and see review below). We acknowledge that plasma may not be a comprehensive representation of all oxidative stress in all tissues (Costantini, 2019), but is recommended for free-ranging animals for which terminal sampling is not appropriate (Monaghan *et al.,* 2009; Costantini, 2019). Sampling multiple tissues would undoubtedly provide a more complete picture, however, we were limited in our ability to sample other tissues and terminal sampling was not an option. Additionally, although factors such as preservation techniques and slight changes in methodology may introduce variability, we compare multiple studies that have collected samples similarly and used the same assays in avian systems. Compared to studies that used similar assays for plasma, our ROM values for Arctic terns and long-tailed jaegers were slightly higher than Scopoli’s shearwater (Costantini and Dell’Omo, 2015), canaries (Costantini *et al.,* 2016) and kestrel nestlings (Costantini and Dell’Omo, 2006). Tern and jaeger ROM values in our study were similar to European starling ROM values (Fowler *et al.,* 2018), and in line with breeding Adelie and Gentoo penguins (Beaulieu *et al.,* 2013), but somewhat lower than foraging Adelie penguins (Beaulieu *et al.,* 2010) and bats (Costantini *et al.,* 2019; Beaulieu *et al.,* 2020). The range of antioxidant values we found is similar to Scopoli's shearwater (Costantini and Dell’Omo, 2015), European starlings (Fowler *et al.,* 2018), bats (Costantini *et al.,* 2019) and breeding Gentoo and Adelie penguins (Beaulieu *et al.,* 2013), but antioxidants in both terns and jaegers in our study were slightly higher than levels found in foraging Adelie penguins (Beaulieu *et al.,* 2010). Arctic tern and long-tailed jaeger uric acid values are comparable to songbirds (European starlings (Fowler *et al.,* 2018) and common blackbirds (Eikenaar *et al.,* 2017)) as well as migratory shorebirds (bar-tailed godwit (Landys *et al.,* 2005). Few other studies have reported oxidative stress in seabirds. Breeding thick billed murres show considerable interannual variability, but minimal differences between males and females in oxidative damage. Interestingly, the uric acid measurements in murres were tremendously higher than our measurements (36000–57 000 micromolar in murres compared to our converted values of ~ 2200 micromolar) (Lin *et al.,* 2022). The authors suggest conditions prior to the breeding season may affect oxidative status. Hoffman *et al.,* 2011 measured the relationship between mercury contaminant load and oxidative stress in Forster’s and Caspian terns, but they used different assays and different tissues (brain, kidney and liver) than this study, preventing direct comparison (Hoffman *et al.,* 2011).

Hematocrit and hemoglobin as indicators of aerobic capacity have been linked to behavioral and ecological parameters in birds (Wagner *et al.,* 2008b; Williams, 2012). For example, foraging efficiency and dive duration of Macaroni penguins was linked to hematocrit (Crossin *et al.,* 2015). Our hematocrit values were a bit lower than songbird (incubating starlings 53% (Fowler *et al.,* 2018) or migratory shorebird (bar-tailed godwit 47–51% (Landys-Ciannelli *et al.,* 2002) hematocrit. However, our values are comparable with those reported for another marine bird and a congeneric of long-tailed jaeger, the great skua, at 45% to 46% (Kalmbach *et al.,* 2004). Our study provides the first records of hematocrit in long-tailed jaegers and for Arctic terns, only hematocrit in chicks has previously been reported (<40%) (Bech and Klaassen, 1996). Previous studies have found differences in hematocrit due to egg production in songbirds (Williams *et al.,* 2004; Wagner *et al.,* 2008a). However, we found no differences between males and females, and without other values to compare to, we assumed they have recovered from any anemia due to egg production by our incubation sampling time period. The only pattern we found with aerobic capacity variables was Arctic tern spring migration rate (km/day) and hematocrit. This strong positive relationship confirms the importance of aerobic capacity for high intensity exercise.

A variety of uncontrolled variables in *in vivo* ecological studies has the potential to obscure clear relationships (Cohen *et al.,* 2012) and in these free ranging birds we were unable to control for many of the parameters suggested by (Hõrak and Cohen, 2010). For example, we could not take repeated samples of the same individual, or control for age (although all birds were nesting adults), diet or activity prior to capture. Controlled, manipulative studies show that age and prior stress can affect oxidative stress (Marasco *et al.,* 2017; Majer *et al.,* 2019). However, our study contributes new information to our understanding of oxidative balance in long distance migrating seabirds. Future studies attempting to detect individual physiological signals of migration should either work on a sampling time frame closer to arrival from migration or attempt to collect additional variables such as age.

The relevance of physiology is not always immediately apparent to conservation practitioners, but the physiological tolerance of organisms to changing environments is important to understanding threats to biodiversity (Visser, 2008; Cooke and O’Connor, 2010; Cooke *et al.,* 2021). We feel our data provide baseline values and some first insights into potential physiological flexibility. Physiological biomarkers, like oxidative stress, have been identified as important to building a ‘tool box’ to predict responses to conservation challenges using physiology (Madliger *et al.,* 2018; Cooke *et al.,* 2021), but the establishment of baselines is an important first step. As we begin to understand more about how environmental changes may affect oxidative challenges for organisms, it is important to determine their capacity to respond to such changes. Cooke *et al.* (2021) identified the potential for physiological tolerance as an important theme in conservation physiology.

Long-tailed jaegers are considered sentinel species of environmental change in Alaska (U.S. Fish and Wildlife Service, 2009), and a substantial proportion of their global population is thought to be in the State, although population estimates are unavailable for most of their Alaskan distribution (U.S. Fish and Wildlife Service, 2009). Tundra breeding jaeger populations are at risk as tundra conversion (encroachment of changing habitat upward into the tundra) occurs (Terskaia *et al.,* 2020; Raiho *et al.,* 2022). Changing plant communities (Elmendorf *et al.,* 2012; Bjorkman *et al.,* 2018), alterations in the small rodent populations (prey for long-tailed jaegers (Gilg *et al.,* 2003; Barraquand *et al.,* 2014)) and expanding ranges for other avian species (Elmhagen *et al.,* 2015) are just some of the shifts associated with climate change. Thus, new challenges related to predation, competition and exposure to new physiological and ecotoxicological stimuli such as pollutants are likely to present themselves (Gilg *et al.,* 2012). Physiology (including stress response and immunity) have been shown to mediate avian populations' resilience to disturbance in forest communities (Messina *et al.,* 2018). Thermal challenges (for example, increased temperatures) are also likely to cause oxidative stress in animals (Andersson *et al.,* 2018; Beaulieu *et al.,* 2020).

Management frameworks, such as the climate change vulnerability assessment (CCVA), have emerged to attempt to consolidate many variables affecting organisms in order to assess components such as exposure, sensitivity, and adaptive capacity (Foden *et al.,* 2019; Thurman *et al.,* 2022). The adaptive capacity refers to the ability of a species to adjust to climate change and includes data on 36 different attributes; among them data on physiological tolerance (Thurman *et al.,* 2020). Existing knowledge gaps for species is a serious limitation of using an adaptive capacity framework for setting climate informed conservation goals for species (Thurman *et al.,* 2020). Managers attempting to predict whether species may be vulnerable to projected climate scenarios must have data available on these attributes. Our data do not provide a silver bullet to predicting how jaegers and terns will be affected by climate change, but our data suggest a hypothesis that better integration in terns indicates that terns are more able to manage increases in oxidative challenges. As climate change presents environmental challenges that are likely to lead to increases in oxidative stress, terns maybe more flexible to accommodate these changes, while jaegers may be less able to respond to the physiological challenges that will likely accompany environmental change. We feel our study contributes data that could be used in management frameworks such as CCVAs and physiological tolerances, enabling managers to better prioritize threats and potential responses of tundra breeding birds.

Additionally, as we provide the first measurements of oxidative physiology in these species, we provide baseline data that conservation practitioners can use as a reference point to compare subsequent oxidative physiology values in terns or jaegers. As future population trends are monitored, in concert with oxidative physiology values, this will further inform our understanding of oxidative physiology as a biomarker and its relationship to demographic parameters. Although there are always caveats with baseline data due to the difficulty of sampling free ranging animals, our data provide the first measurements as the Arctic environment is expected to change rapidly with climate change (IPCC, 2021). Our hope is that the knowledge about potential vulnerabilities will prove useful as conservation practitioners must incorporate many variables in a complex decision-making process that requires prioritization among species and habitat management decisions in the face of rapid environmental change. If the scientific community hopes to detect oxidative stress responses to accelerating climate change in seabirds that incubate in Arctic and alpine tundra, we must start to contribute to longitudinal data sets for future comparisons.

## Supplementary Material

Supplementary_Material
