# Supplementary material for: Oxidative physiology of two small and highly migratory Arctic seabirds: Arctic terns (Sterna paradisaea) and long-tailed jaegers (Stercorarius longicaudus)
Source: Conserv Physiol. 2023 Aug 19;11(1):coad060. doi: 10.1093/conphys/coad060 (PMC10616233; doi:10.1093/conphys/coad060)
Supplement: Supplementary_Material [file supplementary_material.pdf]

Supplemental Figures

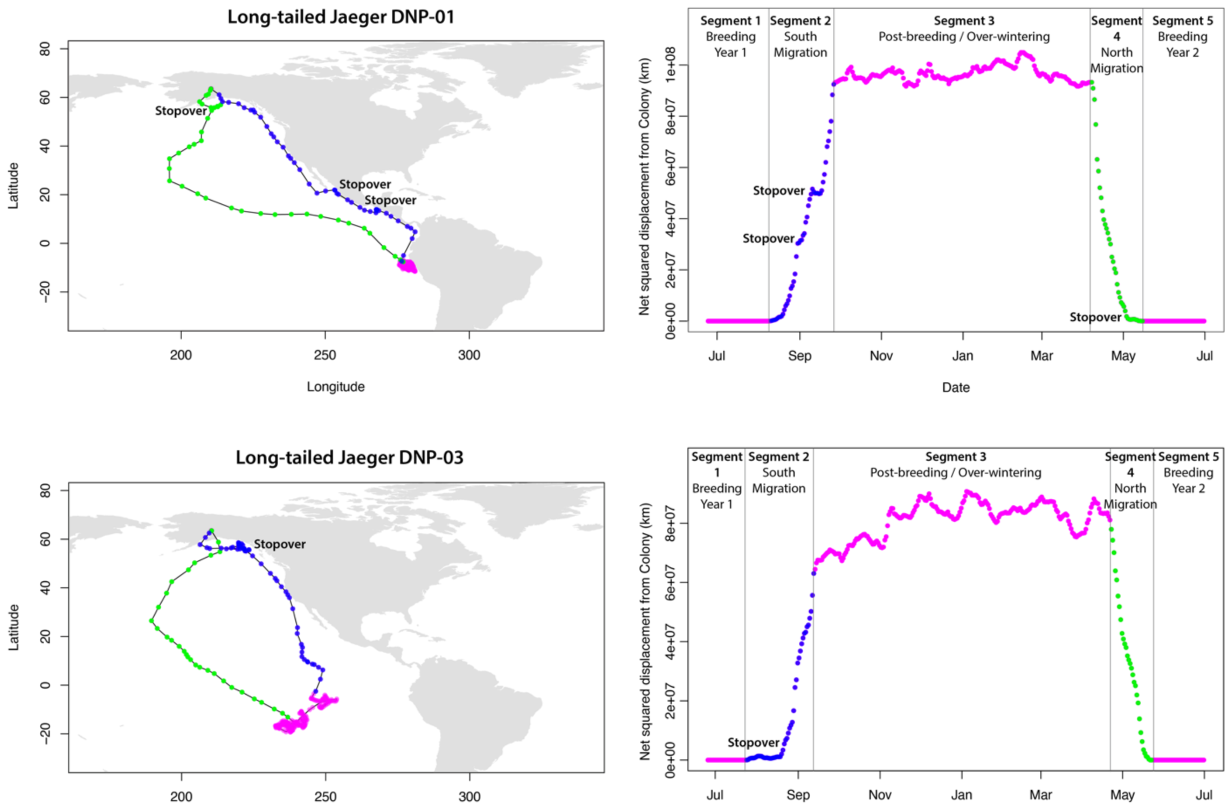

Figure S1: Identification of periods of the annual cycle of migrating Long-tailed Jaegers tracked with electronic tags in Alaska, USA (left plots) based on the change in time of net squared displacement (squared daily distance from nest site, right plots). Migratory periods are indicated by a steep slope and periods of residence by a stable slope. Segments were demarcated by an automatic change point analysis (*changepoint* package in program R) and then manually assigned to a period of the annual cycle based on date.

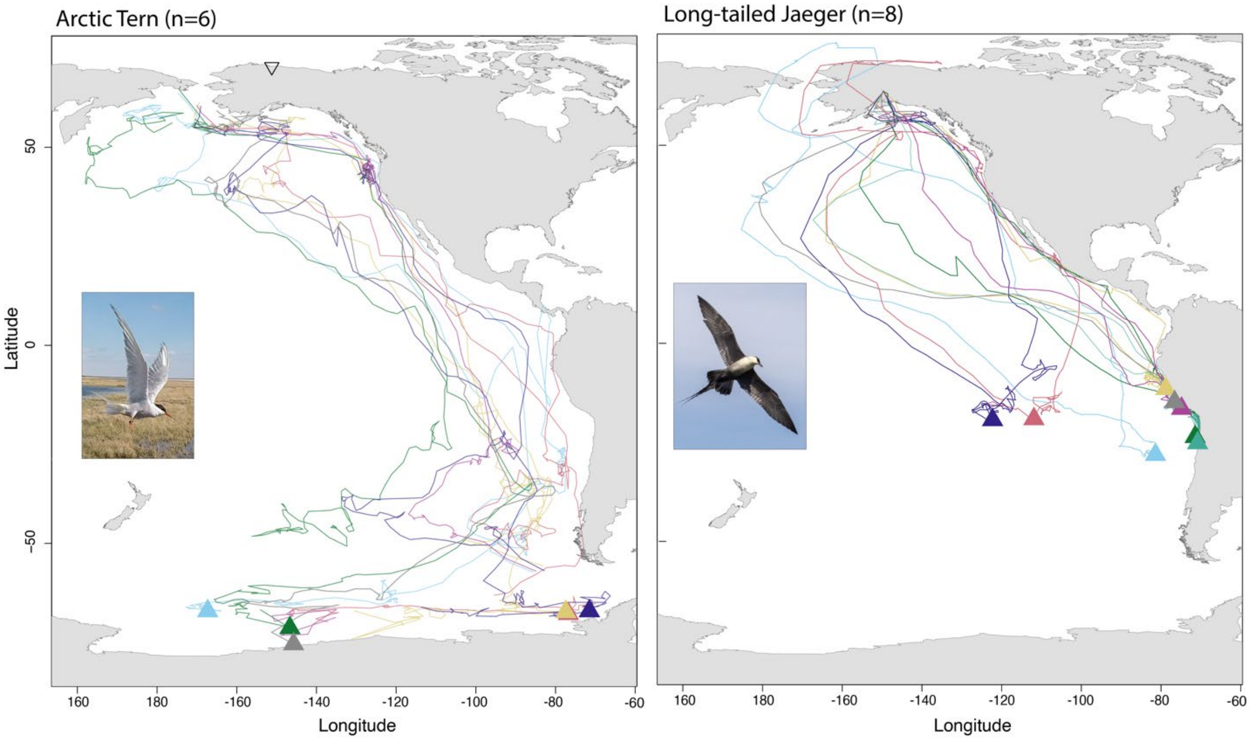

Figure S2: Full-annual-cycle movement paths of Arctic Terns (n=6) and Long-tailed Jaegers (n=8) tracked with electronic tags from nesting grounds in Alaska, USA. See methods for deployment and track processing details. Arctic Tern paths previously published in Wong et al. 2022. Triangles indicate location birds reached the maximum distance from colony. Colors indicate individual id as in Figure 1.
